# Sun protection knowledge and behaviors of agricultural workers in Turkey: a cross-sectional study

**DOI:** 10.1186/s12889-024-20121-8

**Published:** 2024-09-20

**Authors:** Elif Uner Asil, Ayşe Dagli, Ozcan Aygun

**Affiliations:** 1 Fethiye Faculty of Health Science, Public Health Nursing Department, Mugla Sitki Kocman University, Calica Mevkii/Karaculha, Fethiye/Mugla, Turkey; 2 Institute of Health Sciences, Department of Nursing, Public Health Nursing Master’s Program, Ali Koçman Kültür Sanat Sitesi, Zemin Kat, Kötekli / Mugla, 48000 Turkey

**Keywords:** Agrıculture, Skin cancer, Skin cancer knowledge, Sun protection, Sun protection behaviors

## Abstract

**Background:**

Agricultural workers are at risk of developing skin cancer due to prolonged exposure to the sun during their daily work. This study was conducted to determine sun protection knowledge and behaviours of agricultural workers in Turkey.

**Methods:**

The cross-sectional study was conducted with 460 participants working in agriculture. The data were collected using a sociodemographic form, Skin Cancer and Sun Knowledge Scale and Sun Protection Behaviour Scale. The data were analysed using One Way ANOVA and Independent Samples t Test.

**Results:**

Participants mean total score on the Skin Cancer and Sun Knowledge Scale was 15.24 ± 2.47 (max-min 0–25) and the mean total score on the Sun Protection Behaviour Scale was 24.10 ± 4.46 (max-min 8–40). Statistically significant disparities were observed between the SCBS, SPBS and their sub-divisions along with the working period, age, marital status, gender, level of education, income status, skin type and agricultural working status of the participants (*p* < .05).

**Conclusion:**

The study found that people working in the agriculture had inadequate sun protection behaviours and knowledge. Based on the study’s results, it is proposed to create intervention programmes that specifically target single, male, middle-aged or older individuals with extended working hours and low levels of education and income.

## Background

Skin cancer is a major health problem caused by several complex interrelated factors. The incidence of skin cancer is increasing worldwide [1]. Exposure to sunlight is the single most important environmental factor that increases the likelihood of developing skin cancer [2]. As the ozone layer continues to deplete, more ultraviolet (UV) radiation is expected to reach the Earth’s surface. This will increase the harmful effects of sun exposure on health, increasing the risk of skin cancer and other UV-related health problems. Furthermore, several studies have shown that individuals’ sun protection practices and awareness of skin cancer risk are insufficient in their daily lives [3–6]. People who work in certain occupations, particularly those who are exposed to the sun for prolonged periods of time without protection, have an increased risk of developing skin cancer [7]. One such group is agricultural workers.

Agricultural workers are often exposed to prolonged sun exposure while working in open fields or greenhouses. Several studies suggest that the perception of risk and knowledge of sun protection measures among agricultural labourers is inadequate, as are their sun protection practices. It has been specifically noted that older individuals with lower levels of education who have worked in agriculture for over a decade and possess inadequate perceptions, knowledge, and behaviours towards sun protection, pose a considerable risk of suffering from sunburn and skin cancer [8, 9]. It has been suggested that agricultural workers may have a higher risk of various skin problems and skin cancer because of their profession [10–13]. Studies have also shown that agricultural workers have a higher incidence of skin cancer compared to the general population [14–17].

Owing to its geographical setting, solar radiation levels in southern Turkey intensify between April and October. In these regions, the average UV index registers between 8 and 10, indicating high and hazardous levels [18]. As a result, it is crucial to identify the extent of sun protection awareness and practices amongst agricultural workers in Southern Turkey to safeguard both their health and sun safety [10, 19]. The purpose of this article is to determine the sun protection knowledge and behaviors of agricultural workers in Turkey.The research questions of this study are as follows:

What are the knowledge levels of agricultural workers about skin cancer, sun health and sun protection behaviors?

What are the sociodemographic factors that affect the knowledge levels of agricultural workers about skin cancer, sun health and sun protection behaviors?

## Methods

### Sample and design

The sample of the cross-sectional study consisted of workers in Fethiye district of Mugla province, where greenhouse agricultural workers work intensively. The eligibility criteria of the participants who participated in this research are; being between the ages of 18–65, working in the agricultural sector, agreeing to participate voluntarily after understanding the purpose of the research explained by the researchers and signing the informed consent form in writing, agreeing to fill in the data collection forms. During the research, probability simple random sampling method was used. To determine the required sample size, a power analysis was performed using the mean scores on the Skin Cancer and Sun Knowledge Scale from a previous study conducted in this population [20].

### Ethical considerations

Ethical approval for the study was obtained from Muğla Sıtkı Koçman University Health Sciences Ethics Committee (19/01/2023-220206). In addition, participant consent was obtained from all participants in the study. In addition, permission to use was obtained for the measurement tools used in the study. All stages of the study were conducted in accordance with the provisions of the Declaration of Helsinki.

### Data collection

The data were collected by the researchers through face-to-face interviews. The reason face-to-face interviews were preferred is that there may be individuals with low literacy or low education levels among agricultural workers. This may create difficulties in accessing and understanding online self-report questionnaires. Face-to-face interviews eliminate such obstacles and ensure more reliable data collection. In addition, face-to-face interviews encourage participants to participate in the research and achieve higher response rates. It took an average of 10 min to fill in the forms used during the interviews.

### Measurements

The study’s data was obtained through a sociodemographic form, the Skin Cancer and Sun Knowledge Scale, and the Sun Protection Behaviour Scale.

### Sociodemographic form

The form consists of a total of eleven questions, including sociodemographic characteristics of the participants and questions about skin cancer [21]. These variables were selected to understand the demographic and occupational characteristics of the participants and to examine the effects of these characteristics on sun protection knowledge and behaviours.

### Skin cancer and sun knowledge scale

The Skin Cancer and Sun Knowledge Scale, developed by Day et al. in 2014, was designed to measure the level of knowledge of individuals about skin cancer and sun protection. The reliability of the scale is adequate according to the KR-20 coefficient (KR-20 = 0.69) [22]. The Turkish validity and reliability study of the scale was conducted by Öztürk Haney et al. in 2018. The content validity index (CVI) for the Turkish Skin Cancer and Sun Knowledge Scale was determined as 93.71%. The KR-20 internal consistency reliability coefficient of the scale was found to be 0.51, and the test-retest reliability at two-week intervals was 0.52 [23]. In this study, the internal consistency coefficient (KR-20) of the scale was calculated as 0.47.

The scale includes subjects such as skin cancer risk factors, skin cancer symptoms and sun protection methods. The scale, which consists of a total of twenty-five questions, gives 1 point for each correct answer and 0 points for incorrect or blank answers. Total scores vary between 0 and 25 and help to determine the level of knowledge of individuals about skin cancer and sun protection (0–8 points: Insufficient level of knowledge, 9–16 Points: Moderate level of knowledge, 17–25 points: Important level of knowledge).

### Sun protection behaviour scale

The scale was developed by Maddock et al. (2005) to measure the frequency of sun protection behaviours of individuals [24]. This scale is used to measure the frequency of sun protection behaviours ranging from never to always (1–5) when staying outside for more than 15 min. The scale consists of a total of 8 items. Reliability and validity analyses of the Turkish version of the scale were conducted by Aygün and Ergün (2015) and Cronbach’s alpha coefficient was found to be 0.78 [21].

The lowest score that can be obtained from the scale is 8 and the highest score is 40. The scale consists of three subscales: avoiding sunbathing, using sunscreen, and wearing a hat. The total mean scores of the subscales vary between 3 and 15 for sunbathing avoidance, 3–15 for sunscreen use and 2–10 for hat use. In the study, the overall Cronbach’s alpha coefficient of the scale was calculated as 0.71, while the Cronbach’s alpha coefficients of the subscales ranged between 0.81 and 0.88.

### Data analysis

As a result of the analysis made with GPower 3.1.9.7 software, it was calculated that the study needed a total of 388 participants to provide 80% power with a 95% confidence interval and 5% sampling error [25]. In order to prevent any sample loss, the sample size was increased by 20% and 466 participants were planned to be included in the study. As a result, a total of 460 participants working in agriculture, between the ages of 18–65, who were approved to participate in the study, constituted the sample of this study.

The data analysis was conducted using IBM SPSS 22.0 package programme. Frequency, percentage, and normal distribution tests were utilized to analyze the data. To compare mean scale scores and independent variables, The data were analysed using One Way ANOVA and Independent Samples t test. Statistical significance level was accepted as *p* < .05 in the initial tests used in the study. The Bonferroni correction was applied to statistically significant comparisons as a result of a one-way analysis of variance.

In the study, the Fitzpatrick skin type classification, which is widely used to determine the skin type of the participants, was used. This classification is based on how the skin reacts to sun exposure and includes six main skin types; Type I: Very fair skin, always burns, never tans, Type II: Light skin, usually burns, difficult to tan, Type III: Medium skin, sometimes burns, tans slowly, Type IV: Olive skin, rarely burns, tans easily, Type V: Brown skin, very rarely burns, tans very easily, Type VI: Black skin, does not burn, tans very dark [26].

## Results

### Sample

The number of individuals who were examined and confirmed to be eligible according to the eligibility criteria determined in the study is 466 in total. 460 of these individuals agreed to participate in the study and were included in the data collection process. In this case, the participation rate was calculated as (460/466) ×100 ≈ 98.72%. The reason 6 missing data could not be collected is that the participants could not spare time for the interview due to busy working hours or personal reasons. Of the participants, 32.7 per cent were between the ages of 30–39, 62.7 per cent were female and 34.2 per cent were primary school graduates. The rate of self-employed was 46.7% and many of them worked between 1 and 8 h a day on average. 57.8% of the participants had sensitive skin type and 81.1% had experienced sunburn more than three times (Table 1).

**Table 1:** Distribution of socio-demographic characteristics of participants

Variable	Number	%
**Age group**		
20–29 ages	95	21.1
30–39 ages	147	32.7
40–49 ages	116	25.8
50 and older	92	20.4
**Gender**		
Female	282	62.7
Male	168	37.3
**Marital status**		
Married	369	82.0
Single	81	18.0
**Education level**		
Literate	37	8.2
Primary School	154	34.2
Middle School	88	19.6
High School	145	32.2
University	26	5.8
**Perceived ıncome level**		
Income equals expenditure	186	41.3
Income less than expenditure	142	31.6
Income more than expenditure	122	27.1
**Employment status in agriculture**		
Self-employed	210	46.7
Casual labourer/daily worker	23	5.1
Family worker	199	44.2
Seasonal workers	18	4.0
**Daily working hours**		
1–8 h	240	53.3
8 h over	210	47.7
**Skin type**		
Sensitive	268	57.8
Normal	168	37.3
Dark	22	4.9
**Number of sunburns**		
Less than 3 times	85	18.9
More than 3 times	365	81.1

### Level of knowledge and behaviour

Participants’ mean total score on the Skin Cancer and Sun Knowledge Scale was 15.24 ± 2.47 (max-min 0–25) and the mean total score on the Sun Protection Behaviour Scale was 24.10 ± 4.46 (max-min 8–40) (Table 2).

**Table 2:** Skin cancer and sun information scale. Mean scores of the Sun Protection Behaviour Scale and its subscales

Scales	*n*	Mean	SD	%95 CI		Median	IQR	Skewness	Kurtosis
				Lower	Upper				
SA information	450	15.24	2.47	3.95	4.24	15.00	3	0.04	-0.34
Avoiding the sun	450	12.36	2.40	12.14	12.58	12.00	4	-0.54	-0.52
Using sunscreen	450	4.44	2.16	4.24	4.64	3.00	3	1.81	4.34
Using a hat	450	7.29	2.24	7.08	7.50	7.00	4	-0.44	-0.54
TOTAL	450	24.10	4.46	23.69	24.51	24.00	6	-0.88	0.80

n = Number. SD = Standard Deviation. IQR = Interquartile Range

### Factors associated knowledge

The mean scores for individuals working more than eight hours a day (t=-2.99, *p* = .003) were found to be significantly higher than those for individuals working one to eight hours a day. (Table 3). The skin type (*p* < .001) were found to have a statistically significant effect on the total scores of the Skin Cancer and Sun Knowledge Scale (Table 3). The results indicated that the mean score for the Skin Cancer and Sun Knowledge Scale was significantly higher for workers with both sensitive (*p* < .001) and dark skin types than for those with normal skin type.

**Table 3:** Comparison of knowledge- skin Cancer and Sun Knowledge Scale score averages according to Sociodemographic characteristics of the participants

Variable	*n*	Mean	SD	Median	Test	*p*	Bonferroni adjustment
**Age group**							
20–29 ages	95	15.55	2.64	15.00	F = 2.39	0.067	
30–39 ages	147	14.96	2.80	15.00			
40–49 ages	116	15.00	2.10	15.00			
50 and older	92	15.66	2.09	15.00			
**Gender**							
Female	282	15.16	2.42	15.00	t = 0.45	0.381	
Male	168	15.37	2.56	15.00			
**Marital status**							
Married	369	15.11	2.28	15.00	t= -1.91	0.059	
Single	81	15.82	3.18	15.00			
**Education level**							
Literate	37	14.70	1.66	15.00	F = 1.42	0.224	
Primary School	154	15.19	2.54	15.00			
Middle School	88	15.00	2.54	15.00			
High School	145	15.45	2.43	15.00			
University	26	15.92	2.95	17.00			
**Perceived ıncome level**							
Income equals expenditure	186	15.38	2.59	15.00	F = 1.19	0.304	
Income less than expenditure	142	14.97	2.37	15.00			
Income more than expenditure	122	15.32	2.40	15.00			
**Employment status in agriculture**							
Self-employed	210	15.20	2.45	15.00	F = 0.65	0.582	
Casual labourer/daily worker	23	15.43	0.84	15.00			
Family worker	199	15.18	2.70	15.00			
Seasonal workers	18	16.00	1.08	16.00			
**Daily working hours**							
1–8 h	240	14.92	2.60	15.00	t= -299	0.003**	
8 h over	210	15.60	2.27	16.00			
**Skin type**							
Sensitive^1^	268	4.21	1.43	16.00	F = 15.60	< 0.001***	1 = 3 > 2
Normal^2^	168	3.86	1.59	14.00			
Dark^3^	22	4.54	1.71	16.00			
**Number of sunburns**							
Less than 3 times	85	3.82	1.53	16.00	t= -1.26	0.165	
More than 3 times	365	4.16	1.51	15.00			

n = Number, SD = Standard Deviation, F = One Way ANOVA, t = Independent Samples t test,

*** *p* < .001, ** *p* < .01, *p < .05

In the Bonferroni correction, the compared groups were numbered with metadata (1, 2, 3, etc.) and each group was compared within itself

### Factors associated behaviour

The mean scores for sunscreen use and sun avoidance (*p* = .015) showed a statistically significant increase for women compared to men (*p* = .011) and for married compared to single individuals (*p* < .001) (Table 4). A statistically significant difference was found between the employees’ age groups and their mean scores on the Sun Protection Behaviour Scale, as well as the sun avoidance and sunscreen use subscales (*p* < .001) (Table 4). The mean sun avoidance scores of workers in the 20–29 age group were found to be significantly higher than those in the 30–39, 40–49, and 50 + age groups (*p* = .002, *p* < .001, *p* < .001, respectively). Furthermore, the mean scores of sun screen use among workers in the 20–29 age group exhibited a statistically significant difference compared to those in the age groups 30–39 (*p* = .001), 40–49 (*p* < .001) and 50 years and over (*p* < .001). Additionally, the mean score for sunscreen use among workers in the 30–39 age group was significantly higher than that of workers 50 years of age and above (*p* = .001). Finally, it was determined that the mean scores of the Sun Protection Behaviour Scale of workers in the 20–29 age group were significantly higher than those in the 30–39, 40–49 and 50 years and over age groups (*p* < .001).

**Table 4:** Comparison of the Mean scores of Sun Protection Behaviour Scale and its Subscales according to the Sociodemographic characteristics of the participants

Variables	*n*	Sun Avoidance			Sunscreen Use			Hat Use			SPBS total		
		Mean	SD	Median	Mean	SD	Median	Mean	SD	Median	Mean	SD	Median
**Age group**													
20–29 ages^1^	95	13.38	2.04	15.00	5.58	2.80	6.00	7.76	2.12	8.00	26.74	4.24	26.00
30–39 ages^2^	147	12.25	2.42	13.00	4.53	1.97	3.00	7.21	2.27	7.00	24.00	4.63	24.00
40–49 ages^3^	116	12.12	2.19	12.00	4.17	1.87	3.00	7.18	2.22	7.00	23.47	3.76	24.00
50 and older^4^	92	11.80	2.69	12.00	3.45	1.32	3.00	7.06	2.31	7.00	22.32	4.03	23.00
Test		F = 8.29			F = 17.81			F = 1.90			F = 18.75		
p		< 0.001***			< 0.001***			0.129			< 0.001***		
**Bonferroni adjustment**		1 > 2 = 3 = 4			1 > 2 = 3 = 4 2 > 4						1 > 2 = 3 = 4		
**Gender**													
Female	282	12.39	2.52	12.00	4.64	2.32	3.00	7.14	2.32	7.00	24.19	4.86	24.00
Male	168	12.31	2.20	12.00	4.10	1.80	3.00	7.53	2.08	7.50	23.95	3.70	24.00
Test		t = 0.36			t = 2.79			t= -1.77			t = 0.059		
p		0.719			0.006**			0.077			0.551		
**Marital status**													
Married	369	12.47	2.40	12.00	4.24	1.89	3.00	7.24	2.24	7.00	23.97	4.33	24.00
Single	81	11.85	2.37	12.00	5.34	2.93	3.00	7.50	2.23	8.00	24.70	5.01	24.00
Test		t = 2.13			t= -3.22			t = − 0.94			t= -1.33		
p		0.033*			0.002**			0.346			0.188		
**Education level**													
Literate^1^	37	12.00	3.67	15.00	3.16	0.68	3.00	6.81	2.73	7.00	21.97	5.16	24.00
Primary School^2^	154	11.96	2.29	12.00	3.56	1.43	3.00	7.22	2.08	7.00	22.75	3.55	23.00
Middle School^3^	88	12.19	1.92	12.00	4.81	2.26	3.00	6.95	2.49	7.00	23.96	4.71	25.00
High School^4^	145	12.91	2.33	13.00	5.40	2.51	6.00	7.43	2.12	8.00	25.75	4.43	26.00
University^5^	26	12.76	2.19	12.00	4.84	1.71	5.00	8.73	1.53	10.00	26.34	3.50	26.00
Test		F = 3.54			F = 20.85			F = 3.87			F = 13.62		
p		0.007**			< 0.001***			0.004**			< 0.001***		
**Bonferroni adjustment**		4 > 2			3 = 4 > 1 = 2 5 > 1			5 > 1 = 3			4 = 5 > 1 = 2		
**Perceived income level**													
Income equals expenditure^1^	186	12.45	2.44	12.00	4.40	2.45	3.00	7.47	2.38	8.00	24.32	5.01	24.00
Income less than expenditure^2^	142	12.21	2.36	12.00	3.97	1.66	3.00	7.21	2.18	7.00	23.41	4.03	23.00
Income more than expenditure^3^	122	12.40	2.40	13.00	5.04	2.05	6.00	7.10	2.07	7.00	24.56	3.95	25.00
Test		F = 0.40			F = 8.37			F = 1.09			F = 2.59		
p		0.668			< 0.001***			0.334			0.076		
**Bonferroni adjustment**					3 > 1								
**Employment status in agriculture**													
Self-employed^1^	210	12.31	2.50	12.00	3.98	1.72	3.00	7.32	2.17	7.00	23.63	4.30	24.00
Casual labourer/daily worker^2^	23	13.30	2.07	15.00	3.95	2.16	3.00	7.34	2.49	8.00	24.60	3.10	24.00
Family worker^3^	199	12.54	2.24	13.00	5.07	2.47	5.00	7.39	2.16	8.00	25.01	0.40	25.00
Seasonal workers^4^	18	9.72	1.60	9.00	3.50	1.04	3.00	5.72	3.08	6.50	18.94	4.35	20.00
Test		F = 9.30			F = 10.94			F = 3.13			F = 12.52		
p		< 0.001***			< 0.001***			0.025*			< 0.001***		
**Bonferroni adjustment**		1 = 2 = 3 > 4			3 > 1 = 4			1 = 2 = 3 = 4			1 = 2 = 3 > 4 3 > 1 = 4		
**Daily working hours**													
1–8 h	240	12.47	2.31	12.00	4.87	2.14	5.00	7.30	2.11	7.00	24.65	4.21	25.00
8 h over	210	12.24	2.51	12.00	3.95	2.07	3.00	7.27	2.39	8.00	23.48	4.66	24.00
Test		t = 0.98			t = 4.58			t = 0.15			t = 2.79		
p		0.327			< 0.001***			0.880			0.005**		
**Skin type**													
Sensitive^1^	268	12.68	2.51	13.00	4.57	2.33	3.00	7.26	2.38	8.00	24.52	4.80	25.00
Normal^2^	168	11.98	2.19	12.00	4.36	1.93	3.00	7.33	2.03	7.00	23.68	3.86	24.00
Dark^3^	22	11.54	2.06	12.00	3.50	1.14	3.00	7.27	2.14	7.00	22.31	3.90	23.00
Test		F = 5.81			F = 2.68			F = 0.04			F = 3.71		
p		0.003**			0.069			0.958			0.025*		
**Bonferroni adjustment**		1 > 2									1 = 2 = 3		
**Number of sunburns**													
Less than 3 times	85	12.64	1.70	13.00	4.29	2.08	3.00	7.49	2.09	7.00	24.43	4.08	24.00
More than 3 times	365	12.30	2.53	12.00	4.47	2.17	3.00	7.24	2.27	7.00	24.02	4.54	24.00
Test		t = 1.19			t= -77			t = 0.91			t = 0.76		
p		0.233			0.477			0.360			0.449		

n = Number, SD = Standard Deviation, F = One Way ANOVA, t = Independent Samples t test, *** *p* < .001, ** *p* < .01, *p < .05

In the Bonferroni correction, the compared groups were numbered with metadata (1, 2, 3, etc.) and each group was compared within itself

A statistically significant difference was found between educational level and the mean scores with higher education levels correlating with increased mean scores on the Sun Protection Behaviour Scale and its subscales for sun avoidance (*p* = .005), sunscreen use (*p* < .001), and hat use (*p* < .004) (Table 4). The mean scores of sun avoidance (*p* = .006) of high school graduates were significantly higher than primary school graduates. It was found that the mean scores of sun screen use of literate and primary school workers were significantly lower than those of middle and and high school (*p* < .001). In addition, the mean scores of the university (*p* = .010) level workers were significantly higher than literate. It was also found that the mean scores of hat use the university graduates were significantly higher than those of the literate (*p* = .008) and middle school (*p* = .004) workers. The mean scores of the Sun Protection Behaviours Scale were found to be significantly higher for university and high school graduates compared to both literate and primary school graduates (*p* < .001).

The subscale score for the use of sunscreen products showed a statistically significant increase in relation to perceived income levels (*p* < .001) (Table 4). It was determined that the mean scores of sun screen use of individuals whose income was higher than their expenditure were significantly higher than those whose income was lower than their expenditure (*p* < .001).

The relationship between agricultural employment status and mean scores for sun avoidance, sunscreen use, and the Sun Protection Behaviour Scale was statistically significant (*p* < .001) (Table 4). The mean scores for sun avoidance and the Sun Protection Behaviours Scale for family workers, self-employed and daily workers are statistically significantly higher than for seasonal agricultural workers (*p* < .001). Furthermore, the mean scores of family workers on sunscreen use and the Sun Protection Behaviours Scale were found to be significantly higher than those of self-employed and seasonal workers (*p* < .01).

Lastly, skin type was significantly difference with sun avoidance (*p* = .003) and the overall Sun Protection Behaviour Scale (*p* = .025). It was determined that individuals with sensitive skin type had higher mean sun avoidance scores than those with normal skin type (*p* = .009). However, Bonferroni test revealed no statistically significant difference between the Sun Protection Behavior Scale and skin type (*p* > .016).

## Discussion

The study investigated sun protection knowledge and behaviours among agricultural workers and found that both were inadequate. Previous studies have also reported deficiencies in sun protection behaviours among individuals working in the agricultural sector [8, 27, 28]. Similarly, behaviour and knowledge levels of other individuals working in open areas have also been found to be inadequate [29, 30]. In addition, in this study, the scores of agricultural workers were found to be lower than those of the general population [31, 32]. This widespread inadequacy in sun protection behaviour among agricultural workers is a major concern for health and safety. The results of the study suggest that agricultural workers should develop more effective strategies to protect themselves from sun-induced hazards.

Participants who worked 8 h or more per day had higher knowledge of sun protection. This suggests that prolonged sun exposure can increase awareness of sun damage risks. Earlier research has likewise indicated that people working in open spaces can be exposed to the sun for 8 h during work [33, 34]. Another study highlighted that daily work hours had no impact on employees’ knowledge and practices of sun protection [35]. This finding indicates that specific occupational groups or geographical factors may be influential in this association. The sun exposure conditions that are distinct to occupational groups or climatic variations in geographic areas can impact employees’ awareness of sun protection.

The study found that participants with sensitive or dark skin types showed a correlation with their scores in sun protection knowledge. Based on these findings, it is recommended that sun protection education and awareness campaigns should be tailored to individuals with diverse skin types to improve effectiveness. Similar research has indicated that individuals with fair skin and a tendency to burn easily, and with sensitive skin type, tend to exhibit higher sun protection scores. Our study supports these findings [27, 36]. Comprehending the impact of skin type on sun protection conduct can promote heightened awareness among individuals to safeguard their skin health and more effective protective measures.

In this study, it was found that young individuals gave more priority to sun protection behaviours and applied protective measures more frequently. The high use of sunscreen and hats in young individuals in the studies conducted is like our study [20, 37]. Young individuals have the potential to encourage sun protection behaviours due to their interest in beauty and skin care. Since the negative effects of the sun on skin health often have long-term consequences, it is important to adopt sun protection habits at a youthful age. Therefore, education and awareness-raising programmes specific to age groups may be an effective strategy to increase sun protection behaviours.

The study found that women had higher sun protection behaviours than men. These results suggest that concerns about skin health and beauty may make women more aware of sun protection behaviours. Other studies [8, 38] have also found that women are more likely to adopt sun protection behaviors. Therefore, the promotion of sun safety education and awareness campaigns can play a crucial role in improving men’s sun safety practices and in reducing the risk of skin cancer.

The study found that sun protection behaviours, such as the application of sunscreen and the use of hats, increased in individuals with higher levels of education. This is like previous studies carried out on agricultural labourers, highlighting that differences in sun protection behaviour are associated with education level and that sun protection behaviour rises with increasing education level [39]. In this context, education programmes are likely to be an effective strategy for the promotion of sun protection behaviour. The development of public health programmes and an increase in awareness-raising activities, particularly for people with low levels of education, may help to reduce the health risks associated with sun exposure.

The study has demonstrated a higher incidence of sunscreen use amongst individuals with a greater income level. This is consistent with prior research indicating that there is a favorable association between adoption of sunscreen use behaviors and higher economic and income levels [40, 41]. In this context, it can be concluded that higher income levels are advantageous in terms of access to and use of sunscreens. The results of this study are also in line with the existing literature.

The research discovered that employment as a seasonal agricultural worker had a negative effect on sun protection behaviour and that sun protection behaviour was inadequate in this group. These results are consistent with the findings of the study, which revealed that sun protection awareness among seasonal agricultural workers is insufficient [42]. In addition, a study of Latino seasonal workers in the USA found that there was a significant gap in their knowledge about skin cancer and sun protection [43]. The study indicates that working conditions and the duration of employment in seasonal agriculture directly influence workers’ sun protection behaviour. Therefore, implementing targeted interventions to improve sun protection behaviours in this population is crucial.

### Limitations and strengths

The results of this study are an indication of the level of awareness and sun protection practices among agricultural workers in a limited area. Therefore, it is not possible to generalise the results of this study to all agricultural workers. In addition, it should be noted that the responses of the participants were based solely on self-reported data, and this remains an inherent limitation of this study. This study represents a rare study of the sun protection knowledge and practices of agricultural workers. The study’s impressive strengths comprise the study’s power (> 80%) and the collection of data from agricultural regions.

The Skin Cancer and Sun Knowledge Scale is a validated instrument in young adults aged 18–26 years and has also been applied to nursing students in Turkey. However, its validity in middle-aged individuals (over 30 years) has not yet been established. This limitation may have some impact on the confidence in the data obtained in this study. To overcome this limitation, future studies should focus on the validity and reliability of the SCSKS in middle-aged individuals. This will provide a more accurate measurement of sun protection knowledge levels in individuals of different age groups.

## Conclusion and recommendations

This study highlights the inadequate sun protection knowledge and behaviors of agricultural workers. The study revealed that age, gender, marital status, education level, income level, employment status, and daily working hours affect sun protection knowledge and behaviors. Many participants exhibited very low levels of sunscreen use behavior. Based on the study results, it is recommended to develop intervention programs targeting individuals who work long hours, are older, single, male, and have low education and income levels.

It is recommended to organize promotional campaigns for sunscreen products and educational programs specifically designed for seasonal agricultural workers. Researchers need to design and conduct intervention studies to improve sun protection knowledge and behaviors among agricultural workers. These programs can be made understandable by supporting them with visual and audio materials. Educational materials should be presented in the local language and cultural context so that everyone can understand. Economic support or subsidies should be provided to increase access to sun protection products (hats, sunscreen, long-sleeved clothing).

Community-based awareness campaigns should be organized in collaboration with local leaders and health workers. These campaigns are important to gain public trust and increase participation. Broad audiences should be reached using media channels such as radio, television and social media. Radio is an effective means of communication, especially in rural areas. Regular health checks and counseling services should be provided to agricultural workers. These services create opportunities for the assessment of knowledge and behaviors regarding sun protection.

## Data Availability

No datasets were generated or analysed during the current study.
